# A Review of the Botany, Traditional Use, Phytochemistry, Analytical Methods, Pharmacological Effects, and Toxicity of Angelicae Pubescentis Radix

**DOI:** 10.1155/2020/7460781

**Published:** 2020-07-31

**Authors:** Liu Yang, Ajiao Hou, Song Wang, Jiaxu Zhang, Wenjing Man, Xinyue Guo, Bingyou Yang, Hai Jiang, Haixue Kuang, Qiuhong Wang

**Affiliations:** ^1^Key Laboratory of Chinese Materia Medica, Heilongjiang University of Chinese Medicine, Ministry of Education, Harbin 150040, China; ^2^School of Traditional Chinese Medicine, Guangdong Pharmaceutical University, Guangzhou 528458, China

## Abstract

Angelicae Pubescentis Radix (AP), as a traditional Chinese medicine (TCM), has been used for thousands of years in China. In this paper, the botany, traditional use, phytochemistry, analytical methods, quality control, pharmacological effects, and toxicity of AP were reviewed. It can provide a reference for the further research and lay a foundation for the rational clinical application of AP. The relevant information on AP was collected from scientific databases (such as Baidu Scholar, CNKI, Google Scholar, PubMed, Science Direct, Web of Science, and SciFinder Scholar), Chinese herbal classics, *Chinese Pharmacopoeia*, PhD and MSc dissertations, and so on. The components which have been isolated and identified in AP include coumarins, volatile oils, organic acids, terpenes, polysaccharides, flavonoids, sterols, and trace elements. Most of them were analyzed by HPLC and GC. A pharmacological study shows that the AP has extensive pharmacological effects, including anti-inflammatory, antirheumatism, sedative and hypnotic, neuroprotection, antioxidation, antitumor, and allergy, and it is widely used in the treatment of the rheumatoid arthritis, knee osteoarthritis, lumbar disc, ankylosing spondylitis, headaches, stroke hemiplegia, Alzheimer's, and arrhythmia. AP is a valuable natural medicinal plant. So far, significant advances have been made in phytochemistry and pharmacology. Some traditional uses have been demonstrated by modern pharmacology. However, the chemical components and pharmacological effects of AP are complex and varied, and there are different standards for the evaluation of its quality and efficacy. The mechanism of action, the structure-activity relationship among the components, and the potential synergistic and antagonistic effects remain to be studied. At the same time, there are few studies on the specific compounds related to its pharmacodynamics. In order to better develop and utilize AP, we should establish a more reasonable, reliable, and accurate quality control standard and focus on the study of bioactive constituents and the demonstration of their mechanism of action.

## 1. Introduction

Angelicae Pubescentis Radix (AP) is derived from the dry root of *Angelica pubescens Maxim* f. *biserrata* Shan et Yuan, a plant in the Apiaceae family. AP was first published in *Sheng Nong's herbal classic*, which is spicy, bitter, and mild in nature and enters the kidney meridian and bladder meridian exerting the remedial effect [1]. AP was recorded and summarized by each edition of the *Chinese Pharmacopoeia*, with the functions of removing wind and dehumidification, relieving pain in paralysis, and so on. AP was often used to treat rheumatism and headaches caused by dampness and cold [2].

A phytochemical study shows that the main active components of AP include volatile oil, coumarin, organic acids, terpenes, polysaccharides, sterols, and other compounds. And, the pharmacological studies show that AP has anti-inflammatory, antirheumatic, sedative, hypnotic, neuroprotective, antioxidant, antitumor, antiallergic, and other effects, which may be closely related to its complex chemical components [3–10]. In clinical application, AP is often used in combination with other TCMs, which has the functions of dispelling wind and dehumidification, promoting blood circulation and relieving pain, relaxing muscles and activating collaterals, tonifying the liver and kidney, and strengthening tendons and bones [11–15], such as Duhuo-Jisheng Wan, Tianhe Zhuifeng Gao, and Tianma Wan. It is usually used to treat arthritis, lumbago, and cephalgia caused by cold and dampness, blocking collaterals of blood stasis, and deficiency of qi and blood [16–18].

AP, as a famous TCM, has been widely used in China for thousands of years. However, there was no in-depth study on the material basis, target, and mechanism of action of AP. There was no optimal processing technique for AP. And, at present, the toxic components and the related mechanism also have not been clarified. In this study, a total of 551 articles were identified from the database in this study, most of which were excluded due to no mention of botany, traditional use, phytochemistry, pharmacological effects of AP, or duplication. 129 references are included in this review. Compared with other reviews, this study provides a more comprehensive overview of botany, traditional uses, phytochemistry, pharmacological effects, and toxicology, with additional reviews of analytical methods, quality control, and processing. It also emphasizes its possible future development direction, which lays a foundation for a comprehensive understanding of AP, further research and development of new drugs, and expansion of its application in clinical and world markets.

## 2. Botany

The *Chinese Pharmacopoeia* (2015 edition) records AP derived from the dry root of *Angelica pubescens* Maxim, f. *biserrata* Shan et Yuan. In early spring, when the seedlings are just germinating, or in late autumn, when the stems and leaves are withered, the soil is digged, the fibrous roots and sediments are removed, and then the roots are baked to half dry, piled up for 2 to 3 days, putting to soft ,and then baking to dry. DH is mainly distributed in Sichuan and Hubei provinces of China [2]. Native plants grow on the dank hillside, grass, or sparse thickets, like in a cool and humid climate, and are cold resistant, and most native plants grow in the altitude of 1200∼2000 meters of the cold highland area. The plant is suitable to grow in fertile, loose alkaline soil, yellow sand soil, or black oil soil but not suitable to grow in the shallow soil, water, and clay soil [19]. There are a variety of invention patents for AP cultivation, planting, and harvesting methods, such as an AP cultivation method (CN. 201911138333) [20], an AP cultivation greenhouse (CN. 201811562359) [21], a method for cultivating high-quality AP (CN. 201810917290) [22], an AP cultivation method for high quality and high yield (CN. 201811053800), and also [23].

The roots of AP are thick and short, slightly cylindrical, 1.5–4 cm in length, and 1.5–3.5 cm in diameter. The lower roots have several curved branches, 12–30 cm in length, and 0.5–1.5 cm in diameter. The root surface is rough, gray-brown, with irregular longitudinal wrinkles and transverse cracks, and has many transverse long lenticels and fine roots [24]. The root head has a ring stripe and polytropic ring petiole mark, and the hollow stem mark is in the middle. Texture is hard, cross section is sallow white, and the cambium is ring brown. The leather part has a brown oil point (tubing), and the wood part is yellow brown. The transverse section of the root has a large curved section and oil spots. AP has a special flavor. It tastes bitter and hot and even can make a tongue numb. AP with thick, oily, and strong aroma are for the best [25].

## 3. Traditional Applications

*Sheng Nong's herbal classic (Han dynasty, 947–950)* records that AP can treat diseases caused by wind and cold, pain caused by bumping of knife wounds, epilepsy, female uterine fibroid hernia, etc. It is recorded in the *Supplementary Records of Famous Physicians* (*the end of Han dynasty, 947–950*) that AP can cure all kinds of limb joint pain caused by wind and evil spirits, whether it is a new disease or a long illness. *Medicinal Theory (Tang dynasty, 923–936)* records that AP can treat the pain caused by wind, cold and wet, qi disorder, itchy skin, pain caused by limb spasm, pain caused by fatigue, and tooth pain. According to *Medical Origins (Jin dynasty, 1186)*, AP can remove moisture and treat headaches and dizziness. *Wang Haogu (Yuan dynasty, 1271–1368)* shows that AP could cure the pain in the waist and leg. According to the book “*Yeyan” (Ming dynasty, 1368–1644)*, AP can be used for sweating, treatment of flank pain, and head and facial pain. *Bencao Tongxuan (Ming dynasty, 1368–1644)* records that AP is used for the treatment of speechlessness, stiff hands and feet, mouth and eyes skewness, swollen eyes, and skin itching. *Bencaozheng (Ming dynasty, 1368–1644)* records that AP can cure rheumatism, foot pain, itching, and stiff limbs. According to *Modern Practical Chinese Medicine*, AP can induce sweating, promote urine discharge, and eliminate edema.

Clinically, AP is often used in combination with other TCMs. The dried root of the TCMs commonly used in combination with AP are as follows:

1. AP compatibility with *Paeoniae Radix Alba*, which has the functions of tonifying liver and dispelling wind [11].
2. AP compatibility with *Dictamni Cortex*, which has the functions of dispelling wind and dehumidification [11].
3. AP compatibility with *Asari Radix et Rhizoma*, which has the functions of dispelling wind and dehumidification, dredging paralysis, and relieving pain [11].
4. AP compatibility with *Taxilli Herba*, which has the functions of dispelling wind and dehumidification, tonifying kidney, and dredging paralysis [12].
5. AP compatibility with *Rehmanniae Radix*, which has the functions of tonifying liver, kidney, and blood, strengthening tendons and bones, tonifying yin, and promoting fluid production [12].
6. AP compatibility with *Angelicae sinensis Radix*, which has the functions of tonifying blood and dispelling wind [12].
7. AP compatibility with *Typhonii Rhizoma*, which has the functions of dispelling wind, dehumidification, relaxing muscles and activating collaterals, and relieving pain [12].
8. AP compatibility with *Ligustici Rhizoma et Radix*, which has the functions of dispelling wind and dehumidification, dissipating cold, and relieving pain [12].
9. AP compatibility with *Schizonepetae Herba*, which has the functions of dehumidification and relieving spasm, relaxing muscles, and activating collaterals [12].
10. AP compatibility with *Ephedrae Herba*, which has the functions of dispelling wind and removing fever, dehumidification, and relieving pain [12].
11. AP compatibility with *Notopterygh Rhizoma et Radix*, which has the functions of dispelling wind and dehumidification, dredging paralysis, and relieving pain [13, 14].
12. AP compatibility with *Gentianae Macrophyllae Radix*, which has the functions of dispelling wind and dehumidification, dredging paralysis, and relieving pain [15].
13. AP compatibility with *Phellodendri Amurensis Cortex*, which has the functions of dehumidification and antipyretic [15].

The commonly used clinical prescriptions include Duhuo-Jisheng decoction [26], Duhuo-Cangzhu decoction, Duhuo decoction [27], Duhuo-Xixin decoction [28], and Duhuo pulvis [29]. Among them, Duhuo-Jisheng decoction is most widely used in clinical practice, which has good effects on rheumatoid arthritis, rheumatoid arthritis, knee osteoarthritis, lumbar disc herniation, headache, stroke, hemiplegia, and other diseases. The dosage forms involved include decoction, liquor, tablet, capsule, pill, aerosol, liniment, powder, paste, etc. [2] (as shown in Table 1).

In addition, it is also used in health care, beauty, and other fields. As early as in *Sheng Nong's herbal classic*, it is recorded that “taking AP for a long time has weight loss and antiaging effects.” The AP wine is recorded in *Qianjinfang*, “Medicinal tea for all kinds of diseases” records the AP tea. *Peaceful Holy Benevolent Prescriptions* records the AP-*Ginseng Radix et Rhizoma* wine. General Records of *Holy Universal Relief* records the AP-Angelicae Sinensis *Radix* wine. In recent years, there are several patented inventions in health care and cosmetics. A kind of AP internal injuries fever health tea (CN. 2012103901941) [30], a kind of beauty dispelling wet medicinal wine (CN. 201710879392) [31], a beauty mask cream (201710782640.6), etc. [32].

## 4. Chemical Component

### 4.1. Coumarins

Coumarin compounds is the general name of o-hydroxycinnamic acid lactones with the basic skeleton of benzo *α*-pyranone, which are one of the main components of AP [33]. In addition, *Chinese Pharmacopoeia* (2015 edition) also uses two coumarin components, osthol and columbianadin, as the quality control indexes of AP.

**Figure 1:**
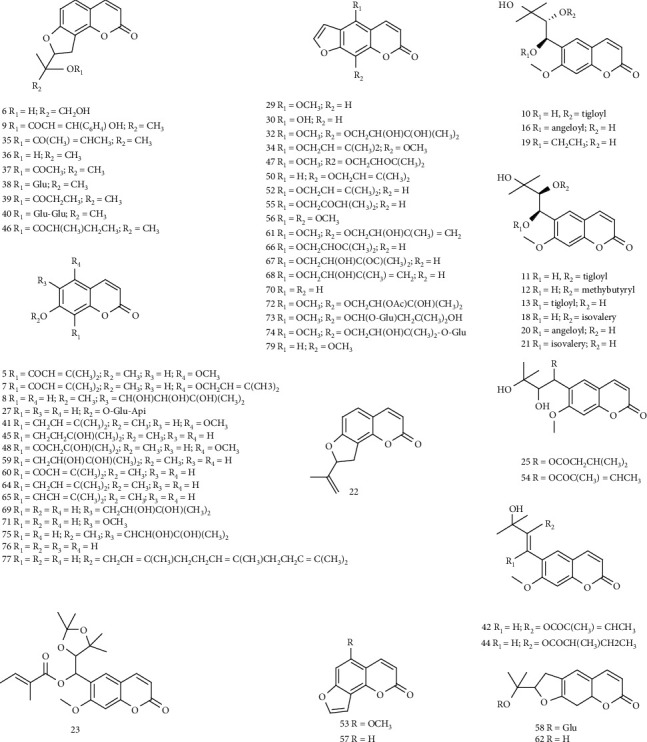
The structures of coumarin compounds of AP.

At present, more than 120 kinds of coumarins have been isolated from various varieties of AP, including furanocoumarins [34], pyranocoumarins [35], dicoumarins [35], simple coumarins [36–41], and individual coumarin glycosides [42]. The components of coumarins isolated from AP are listed in Table 2, and the structures are shown in Figure 1.

### 4.2. Volatile Oils

Volatile oil is a group name of volatile oil-like compounds which has aromatic smell and can be distilled with water vapor without being miscible with water [46]. The volatile oil is a kind of mixture with relatively complex compounds. There are a large number of volatile oil components in AP, mainly including terpenoids, aromatic compounds, and aliphatic compounds [44,47–60], which is shown in Table 3. Gao et al. analyzed the volatile oil compounds from the water extract of AP and *Heracleum candicans* Wall ex Dc dried roots by GC/MS and identified 32 and 45 compounds, respectively [62]. Zhang et al. extracted the volatile oil compounds from the water extract of *Heracleum hemsleyanum* Diels dried roots and identified 53 compounds [63]. Wang et al. extracted and compared the volatile oil compounds of the dried roots and rhizomes of AP and *Angelica dahurica* (Fisch) Benth et Hook, and 90 compounds were detected and 52 were identified from AP; 57 compounds were detected from the dried roots and rhizomes of *Angelica dahurica* (Fisch) Benth et Hook and 37 were identified [64]. Yang et al. used steam distillation to extract the volatile oil from AP and detected 229 chromatography peaks by GC-MS and identified 88 compounds [65]. Zhang et al. extracted the volatile oil from the dried roots of *Heracleum lanatum* Michx by different extraction methods and identified a total of 59 compounds [61]. It has been reported that 40% of the small molecular compounds will change in the process of extraction and detection. Therefore, in this section, only list of the separated compounds from volatile oil of AP is discussed [66]. And, the compounds isolated and identified by these research studies are different, and even the same compounds have different content proportions in different varieties of AP, which may be related to the different species, climatic and environment of the cultivation area, soil conditions, processing methods of the production area, extraction facilities, and extraction conditions.

### 4.3. Organic Acids

**Figure 2:**
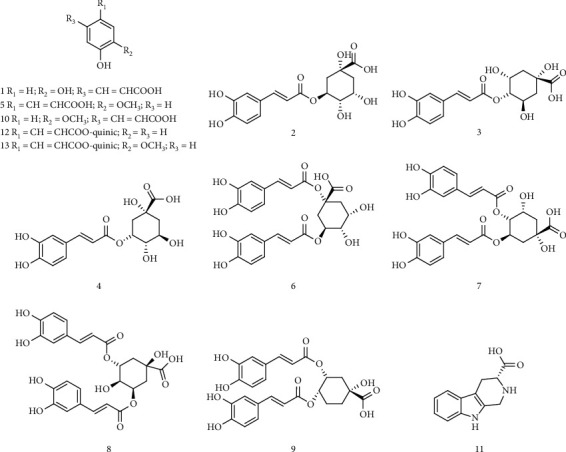
The structures of organic acid compounds of AP.

Organic acid refers to a kind of acid organic compounds containing the carboxyl group, which has a variety of pharmacological effects such as antibacterial, antiviral, antidepression, antidiabetes, antitumor [66], anti-inflammatory [67], antitussive, expectorant [68], and antioxidant [69]. With the first separation of 3-O-trans-coumaroylquinic acid, 3-O-trans-feruloylquinic acid, and other compounds from the methanol extract of the dried roots of AP by Yang et al., some scholars had developed great interest in organic acids compounds in AP [70]. Up to now, the organic acid compounds isolated from AP are far more than these (as shown in Table 4 and Figure 2). We believe that with the progress of time and the development of science and technology, the organic acids in AP will be studied more thoroughly.

### 4.4. Polysaccharides

Polysaccharide compounds have many pharmacological effects, such as antitumor [72], immunomodulatory [73], hypoglycemic [74], hypolipidemic [75], antioxidant [76], and antiaging [77]. Studies have shown that polysaccharides can be obtained from the above-ground parts of AP: 6-O-*β*-D-apiofuranosyl-(1⟶6)-*β*-D-glucopyranosyl scopoletin, 7-O-*β*-D-apiofuranosyl-(1⟶6)-*β*-D-glucopyranosyl umbelliferone, 7-O-*β*-D-glucopyranosyl umbelliferone, 3-O-[*β*-D-galactopyranose-(1⟶2)[*β*-D-xylopyranose-(1⟶4)]-*β*-D-pyranoglucuronic acid]-28-O-*β*-D-pyranogluconoleanolic acid, 3-O-[*β*-D-galactopyranose-(1⟶2)-*β*-D-pyranoglucuronic acid]-O-[*β*-D-galactopyranose-(1⟶2)-*β*-D-pyranoglucuronic acid]-28-O-*β*-D-pyran glucose ivy saponins, 3-O-[*β*-D-galactopyranose-(1⟶2)-*β*-D-pyranoglucuronic acid]-28-O-*β*-D-pyranogluconoleanolic acid, 3-O-[*β*-D-galactopyranose-(1⟶2)-*β*-D-pyranoglucuronic acid] oleanolic acid, 3-O-[*β*-D-xylopyranose-(1⟶4)-*β*-D-pyranoglucuronic acid]-28-O-*β*-D-pyran galactose oleanolic acid, 3-O-[*β*-D-xylopyranose-(1⟶4)-*β*-D-pyranoglucuronic acid]-28-O-*β*-D-pyranogluconoleanolic acid, 3-O-[*β*-D-xylopyranose-(1⟶4)-*β*-D-pyranoglucuronic acid]-hederagenin, 3-O-[*β*-D-xylopyranose-(1⟶4)-*β*-D-pyranoglucuronic acid]-oleanolic acid, 3-O-*β*-D-pyranoglucuronic acid-28-O-*β*-D-pyranogluconoleanolic acid, and 3-O-*β*-D-pyranoglucuronic acid-oleanolic acid [9,42,48].

### 4.5. Others

There are also compounds like adenine riboside, adenosine, allantion, angesesquid A, angesesquid B, 2,3,4,9-cartrahyd-ro-l-H-pyridio[3,4,-b]indole-3-carboxylic acid 1, *α*-caryophyllene, coclaurine, daucosterol, glyceride, glucose, liriodenine, 1-N-methylcoclaurine, oleanic acid, sucrose, *β*-sitosterol, uridine, diterpenic acid, diterpene alcohols, and other compounds and Al, As, B, Ba, Ca, Cd, Co, Cr, Cs, Cu, Fe, Ge, Hg, K, Mg, Mo, Mn, Na, Ni, P, Pb, Se, Sr, Zn, and other trace elements in AP [78].

In this section, the chemical constituents of AP are summarized, which is helpful to further study on the material basis of its efficacy and explore the principle of its prevention and treatment of diseases. And, it also can provide an effective scientific basis for the development of new drugs, the expansion of drug sources, the synthesis of lead compounds or new drugs, the adaptation to clinical use, and the expansion of its market application.

## 5. Analysis Method and Quality Control

There are many kinds of compounds in AP, and their analysis methods are various. And, volatile oil compounds are often detected by gas chromatography-tandem mass spectrometry (GC-MS) [56]. The authors in [79] used the colorimetry method to determine the content of total coumarins in AP. However, the sample preparation of this method is complex and time consuming. Subsequently, thin layer chromatography (TLC) was used to determine the content of columbianetin. Although this method is simple, it is not precise enough and is not ideal for the separation of multicomponent compounds [64]. Some scholars used high-performance liquid chromatography (HPLC) [80–83], high-performance liquid chromatography in tandem with diode array detector (HPLC-DAD) [84,85], high-performance liquid chromatography in tandem with ultraviolet detector (HPLC-UV) [86,87], ultraperformance liquid chromatography-photo diode array detection(UPLC-PDA) [88], and other methods to determine the flavonoids, coumarins, and other compounds with UV absorption in AP. The above methods can analyze the compounds in AP and have good separation effect and stability. However, these methods are not suitable for compounds with no UV absorption or only terminal absorption. There are also scholars who used ultrahigh-performance liquid phase tandem mass spectrometry (UPLC-MS/MS) to determine more than 40 chemical components such as coumarins and phenolic acids in AP. Hou et al. used ultrahigh-performance liquid phase tandem single quadrupole mass spectrometry (UPLC-QDA) to establish the fingerprint of AP, which not only analyzed the type and quantity of the whole compound of AP, but also made up for the deficiency that the above method could not detect compounds with no UV absorption. Wang et al. used high-performance liquid chromatography in tandem with fluorescence detection (HPLC-FD) to determine umbelliferones and scopoletins in AP. The results show that this method is easy to operate, economical, and practical [72]. Yang et al. used ultraperformance convergence chromatography (UPCC) and CO_2_ as the mobile phase to establish the fingerprint of AP, which has the advantages of environmental friendliness, green, and so on [89]. It makes up for the shortcomings of common organic reagents used in the past to pollute the environment [33]. And, other methods are also often used in the detection of coumarin compounds. Among them, HPLC is the one of the most frequently used analysis methods, and it is also the main method for the analysis of other compounds in AP.

In addition, most of these methods have the advantages of rapidness, precision, accuracy, high sensitivity, short analysis time, strong separation ability, good selectivity, simple operation, low detection line, and wide application range. However, most of the methods have the disadvantages of complex sample pretreatment, high-end instruments and equipment, expensive price, use of toxic organic solvents, pollution of environmental soil, not green and environmentally friendly enough, and so on. But, we believe that with the development of science and technology, more and more low-cost and efficient instruments and green reagents will be developed.

The species of AP are complex and difficult to distinguish, which are easily affected by the growing environment, harvesting time, processing methods, and storage conditions. Moreover, the content of active components in AP is different in different areas. Due to these factors, the quality and clinical efficacy of AP cannot be controlled at present.

Currently, the 2015 edition of *Chinese Pharmacopoeia* uses TLC for the qualitative identification of AP and HPLC for the determination of the content of osthol and columbianadin. Moreover, it is stipulated that AP should contain osthol no less than 0.50% and contain columbianadin no less than 0.080%.

However, TCM has the characteristics of multicomponent and multitarget. It is limited to evaluate the quality of TCM only by the content of one or two compounds. In order to evaluate the quality of AP, Yang et al. used the UPLC-PDA method to determine the content of 6 phenolic acids and 7 coumarin compounds [90]. Ding et al. determined the content of coumarin by LC-MS/MS [91]. Yang et al. used the UPLC-MS/MS method to determine the content of 15 compounds in AP and found that columbianetin acetate, osthol, chlorogenic acid, and psoralen were the main components causing the differences in AP from different sources. Hou et al. established the UPLC fingerprint of AP, which again proved that columbianetin acetate and osthol were the main components that caused the difference of AP in different areas [92]. However, there are few methods to evaluate the quality of AP in relation to its pharmacological activity. It is obviously not enough to evaluate AP quality solely on the basis of chemical components. More methods should be established by quality evaluation related to activity or based on chemical activity.

## 6. Processing Method

Processing can remove nonmedicinal parts, keep the active ingredients, make the drug pure, reduce toxicity, detoxify, and change the drug ingredients to enhance the efficacy. *Thunder gong processing theory* (雷公炮炙论) recorded that there was a steaming method of epimedium in the Northern and Southern dynasties. *Lishang Xuduan Fang* (理伤续断方) recorded that there was the method of removing reed in the Tang dynasty. The Song dynasty had to collect the reed and then wash and bake them. *Decoction and Material Medica* (汤液本草) recorded there was a method of peeling and then washing in the Yuan dynasty. In the Ming dynasty, salt water immersion baking was practiced (*Prescriptions for Universal Relief* (普济方)), and also removing section to fry (*Surgical Cases* (外科理例)) and wine wash method (*Curative Measures for All Diseases* (万病回春)). The Qing dynasty has the wine stir-fry (*Chuanyabu* (串雅补)), the wine immersion method (*Gynecology jade ruler* (妇科玉尺)), and so on. Currently, there are net preparation, cut preparation (*2015 edition Chinese Pharmacopoeia*), and frying method (*Integration of Medicine* (医学集成)).

## 7. Pharmacological Effects

As a traditional Chinese medicine, AP has a variety of effects, such as anti-inflammatory [93], anti-rheumatism, sedation [94], hypnosis, neuroprotection [95], antioxidation [96], antitumor [97], and antigastric ulcer [98], which has been studied and confirmed by many scholars. At present, the pharmacological effects of AP are summarized in Table 5, which is described in detail as follows.

### 7.1. Analgesic, Sedative, and Anti-Inflammatory Effect

The decoction of AP has sedative and hypnotic effects on rats and mice and can prevent the convulsive effect of strychnine on frogs but it cannot prevent them from dying. Li et al. studied the anti-inflammatory effect of the AP ethanol extract of different concentrations on cotton ball implantation and found that 60% and 80% ethanol extract had anti-inflammatory effect (*P* < 0.05), while 40% ethanol extract had no such effect [93]. The fingerprint-effect relationship of AP was established by Hou et al., whose research results showed that the extract of AP had significant inhibitory effects on the pain and inflammatory reactions of mice caused by hot plate (*P*< 0.01), acetic acid writhing (*P* < 0.01, the analgesia rate could reach 89.27%), formalin (*P* < 0.01, the analgesia rate could reach 52.91%), ear swelling (*P* < 0.01, the inhibition rate of swelling was up to 69.33%), caused by xylene, and foot swelling (*P* < 0.01, the inhibition rate of inflammation was 23.49%), caused by carrageenan. Even the analgesic and anti-inflammatory activity of AP from some places was stronger than that of the positive drug aspirin. The analysis of fingerprint-effect relationship has shown that osthol, bergapten, columbianetin acetate, and isoimperatorin were the main active components with anti-inflammatory and analgesic effect of AP [92]. However, this study used only a single dose and lacked a dose-dependent study.

Fan et al. studied the analgesic and anti-inflammatory effects of the volatile oil components of AP. The anti-inflammatory effects were observed by the swelling of rats' feet caused by egg white, and the analgesic effects were observed by the hot plate method and acetic acid writhing method in mice. The result showed that the components of volatile oil had no obvious analgesic effect on the pain of mice caused by hot plate, the high dose of the AP volatile oil group could significantly reduce the number of writhing of mice caused by acetic acid (*P* < 0.05, the analgesia rate could reach 76.8%), and the low and high dose of AP volatile oil groups both had a good anti-inflammatory effect on the foot swelling of rats caused by egg white (*P* < 0.01). However, this study only speculated that the anti-inflammatory and analgesic effects of volatile oil in AP were related to the release of 5-HT, and the relevant mechanism was not studied [94].

The study by Qiu et al. found that the ethanol extract of AP had different degrees of inhibition on cyclooxygenase-1 (COX-1) and cyclooxygenase-2 (COX-2). With the increase in the dosage of AP, the inhibitory effect was enhanced, which may also be one of the anti-inflammatory mechanisms of AP [99]. And other studies had shown that AP has analgesic effects by acting on the central and peripheral nervous systems [117]. And, the coumarin compounds in AP could inhibit the increase in peritoneal and skin vascular permeability induced by histamine and bradykinin, suggesting that the anti-inflammatory effect of AP might be related to the inhibition of histamine and bradykinin. Ma established a neural injury model to study the analgesic effect of coumarins in AP, finding that AP has the analgesic effect mainly related to reducing the concentration of proinflammatory cytokines of TNF-*α*, IL-1*β*, and IL-6 in the neural injury model and reduces the expression of TRPV1 and perk in the damaged neurons, which also indicated that it may be related to the presence of osthol and columbianadin in AP [102].

Sun et al. studied the hydrolytic activity of N-acylethanolamine-hydrolyzing acid amidase (NAAA) and its anti-inflammatory effect on the LPS-induced mouse macrophage RAW 264.7 inflammatory response model. The results showed that the volatile oil of AP could inhibit the hydrolysis activity of NAAA and increase the level of N-palmitoylethanolamine (PEA) in RAW 264.7 cells induced by LPS, thereby downregulating the expression of TNF-*α*, iNOS, and IL-6 mRNA and inhibiting the release of TNF-*α* and NO in RAW 264.7 cells to play an anti-inflammatory effect [100]. Li et al. found that angesesquid A and angesesquid B could inhibit the release of nitric oxide (NO) in the inflammatory model cells of chondrocytes of intervertebral disc *in vitro* (*P* < 0.001), thus inhibiting the occurrence of inflammatory response. However, both methods lacked a positive control group [101].

### 7.2. Cardiovascular Effect

The alcohol extract of AP could inhibit the platelet aggregation induced by ADP in rats and the thrombus formed by the Chandler method and could also shorten the length of the thrombus and prolong the time of tail hemorrhage in mice. Moreover, the inhibition rate of platelet aggregation was enhanced with the increase in the AP alcohol extract concentration. When the AP alcohol extract concentration reached 0.4 g/kg, it had an inhibitory effect on the thrombosis formed by carotid vein bypass in rats (*P* < 0.05), and when the AP alcohol extract concentration reached 1.0 g/kg, the inhibition rate could reach 51.1% (*P* < 0.01). However, the method lacked a positive control group and failed to provide reliable data for clinical application. At the same time, only the AP alcohol extract was studied in this study, and there was no research on related bioactive monomer compounds [103].

Some studies had proved that the active ingredients of antiplatelet aggregation and antithrombotic activity were columbianetin acetate, columbianetin, osthol, and columbianedin [118]. Li et al. research showed that gamma-aminobutyric acid (GABA) was one of the main active components of AP, which had antiarrhythmic effect. Gamma-aminobutyric acid (10 mg/kg, iv) could prolong the start time of ventricular arrhythmia induced by aconitine in mice (control group 1.7 ± 0.12 min and GABA group 2.0 ± 0.4 min, *P* < 0.05) and increased the threshold dose (control group 80.9 ± 9.4 g/kg and GABA group 94.3 ± 15.0 g/kg, *P* < 0.05). Gamma-aminobutyric acid (10 mg/kg, iv) could delay the start time of arrhythmias induced by aconitine in rats (control group 3.5 ± 1.1 min, GABA group 13.2 ± 8.8 min, *P* < 0.05), reduce the incidence of ventricular tachycardia (control group 100%, GABA group 50%, *P* < 0.05), shorten the duration of ventricular tachycardia (control group 22.2 ± 6.9 min, GABA group 12.3 ± 0.5 min, *P* < 0.05), and reduce the mortality of ventricular fibrillation (control group 50%, *P* < 0.05). Gamma-aminobutyric acid (10 mg/kg, iv) also had an effect on ventricular action potential in rats, which could reduce APA and APO 50 and APO 90. However, this experiment lacked a positive control group and a comparison between the pharmacodynamics of gamma-aminobutyric acid and the AP crude extract [104].

Chen et al. used Compound Danshen as the positive control group to study the function of activate blood and resolve stasis of the AP alcohol extract. It was found that it can also significantly reduce the whole blood viscosity, plasma viscosity, and red blood cell aggregation index of vertigo patients and increase the velocity of blood flow in cerebral vessels. The results showed that AP alcohol extract treatment was effective, with an effective rate of 95.5%, which was significantly higher than the control group (*P* < 0.05, the effective rate was 79.5%). This showed that the AP alcohol extract could play a good role in activating blood and resolving stasis. However, this study only used a single dose for clinical research, lacking dose-dependent studies and studies on the maximum safe dose of AP [105]. We speculate that the possible mechanism of AP is related to dilation of blood vessels, reduction of blood viscosity, and improvement of microcirculation. It may also be related to GABA contained in AP, increase in cardiac pump function, and influence of the cardiac output. The future scholars should also discuss the specific pharmacodynamic substances that can activate blood circulation and remove blood stasis in the ethanol extract and analyze and verify the pharmacodynamic substances and possible metabolites through *in vivo* and *in vitro* pharmacological experiments, which will be of certain reference value for future studies.

Some studies had also shown that the AP root water extract at low concentration could effectively inhibit the proliferation of human microvascular endothelial cells [97] Chen and Lu showed that AP had a good diastolic effect on vasoconstriction caused by PE and KCl, and its mechanism was related to the influx of CaCl2 [106]. Hu et al. discussed the inhibitory effect and mechanism of AP on angiogenesis and considered osthol as the main component of antiangiogenesis *in vitro*. The inhibition rates of treating with 3.75–30 *μ*g/ml AP extract and osthol for 48 h on cell proliferation of HUVEC were 5.16%–10.15% and 22.64%–65.56% and those of LoVo cells were 2.86%–7.29% and 5.15%–24.39%, respectively. The inhibition rates of treating with 3.75–30 *μ*g/ml AP extract and osthol for 24 h on HUVEC cell migration were between 2.16%–8.00% and 13.70%–63.04%, and the apoptosis rates of HUVEC cells were between 6.1%–14.4% and 18.8%–89.5%, respectively. The results showed that the effect of osthol on the cell cycle was stronger than that of the AP alcohol extract [119], which laid a foundation for AP to become a clinical Chinese medicine for antiangiogenesis.

### 7.3. Neuroprotective Effect

The ethanol extract of AP could improve the learning and memory ability of aging mice by improving the aging of phospholipid components in different parts of the cerebral cortex, hippocampus, and striatum, increasing the concentration of phosphatidylcholine (PC), reducing the content of sphingomyelins (SM), and improving the aging changes of the thymus and hippocampus [94]. It also shown that the mechanism of AP delaying brain aging was related to its antiperoxidation damage of free radicals, reducing the immunosuppression of arachidonic acid metabolites and antagonizing brain inflammation [94]. A study showed that brain aging was related to the increase in malondialdehyde (MDA) content and DNA fragment deletion in brain tissues, and the AP decoction and alcohol extract could reduce the content of MDA and the deletion of the DNA fragment in natural aging mice [96]. Moreover, the effect of the alcohol extract was better than that of the water extract, which may be related to the more effective component coumarin in the alcohol extract [120]. Pei et al. found that the mechanism of the neuroprotective effect of coumarin in AP was related to the inhibition of the content of excitatory amino acid Glu in brain tissues and serum and the inhibition of the expression of the stress factors CHOP and Caspase12 in the substantia nigra in brain tissues [107,108,121]. Li et al. found that AP could significantly improve the activity of mitochondrial respiratory chain enzyme complex I and IV in the brain of aged mice and had a protective effect on the oxidative damage of mitochondrial DNA in mice [109]. Zhang et al. showed that AP could inhibit the expression of IL-1, IL-6, TNF-*α*, p38MAPK, and iNOS in rat brain tissue by regulating NF-*κ*B and increase the ratio of Bcl-2/Bax to reduce the occurrence of inflammation, apoptosis, oxidative stress, and other reactions in brain tissue. AP could also play a neuroprotective role by inhibiting the ability of spleen lymphocytes to secrete proinflammatory factors and increasing the expression of the neurofilament protein [110]. Yu found that the AP root ethanol extract could delay the occurrence of Alzheimer's disease by increasing serum superoxide dismutase (SOD) and reducing acetylcholine esterase (AChE) in brain tissue [111]. Osthol in AP could regulate Jak-SAKT, MAPK, PI3K, TGF-*β*, phosphatidylinositol, and other signaling pathways by participating in the metabolism of glycerol phospholipid, purine, niacin, and nicotinamide. Activation of the PI3K/Akt signaling pathway increases the survival rate of BM-NSCs and inhibits apoptosis to protect BM-NSCs injured by H2O2 [112]. We speculated that after being absorbed into the body, osthol would undergo a series of metabolic reactions to form a lipid soluble metabolite, which would pass through the blood-brain barrier and play a neuroprotective role. But, the metabolomics of osthol at the animal and cellular levels has not been studied. In the future research, the mechanism of action and metabolomics of monomer compounds which play an important role should be studied and analyzed.

By regulating the Notch signaling pathway, BM-NSCs can be promoted to express and differentiate into cholinergic neurons, which can play a role in Alzheimer's disease [114]. And, it played a neuroprotective role by promoting the expression of cyclic adenosine phosphate reactive element binding protein (CREB) and brain-derived neurotrophic factor (BDNF) and increasing the expression of p-CREB and BDNF [114]. However, most of the above studies focused on the crude extract of AP, and there were few studies on the monomer compounds in AP. In the future, monomer compounds should be studied and analyzed, and their unknown mechanism of action in clinical treatment should be clarified.

### 7.4. Antibacterial Effect

It had been reported that AP decoction had significant inhibitory activity on tubercle bacillus and brucella [117]. And, the chloroform extract of *Angelica polyclada* Franch showed inhibition effect on *Bacillus subtilis* and *Escherichia coli*, among which the inhibitory activity against *Escherichia coli* was stronger than that of *Bacillus subtilis* [34]. Zhang et al. used the growth rate method to test the inhibitory effect of the extracts from the petroleum ether layer of AP on the growth of 12 kinds of pathogenic bacteria mycelium. The experimental results showed that the extract had antibacterial effect on the tested bacteria, the experimental results showed that AP had different antibacterial effects on 12 tested fungi, and the inhibition rate of *Rhizoctonia solani* was the strongest, reaching 100%, followed by *Sclerotinia sclerotiorum* and *Phytophthora capsici*, reaching over 80%, while for *Exserohilum turcicum* and *Magnaporthe oryzae*, the inhibition rate was relatively low, and preliminarily identified pimpinellin was the main active component, with strong antibacterial activity [115]. Li et al. isolated and studied the antibacterial active components of the ethanol extract of AP and the results showed that isobergamolactone, sphondin, pimpinellin, and isopimpinellin were the main antibacterial substances, and the EC50 of isobergamolactone, pimpinellin, and isopimpinellin on citrus anthrax was 45 mg/L, among which sphondin had the best inhibitory activity on citrus anthrax, and its EC50 was 41.5216 mg/L [116]. However, the absence of the positive control group and the blank control group and a lack of dose-dependent effects were two limitations of the studies.

### 7.5. Antioxidation Effects

Min *et al.* found that the antioxidant effect of AP was related to the polyphenol compounds contained in it. The study by Chen et al. had shown that the content of malondialdehyde (MDA) in the brain tissues of mice could be reduced by the decoction and alcohol extract of AP, and the effect of the AP alcohol extract was better than that of the water extract, which may be related to the more effective compound coumarin contained in the alcohol extract [96].. Studies by Hou et al. showed that the alcohol extract of AP could inhibit the concentration of MDA in the mouse plasma, and the inhibitory rate could reach 23.49%. Zhang and Du found that AP could delay the occurrence of Alzheimer's disease by increasing the level of serum superoxide dismutase (SOD) [111]. And, Lu and Zhang showed that the ethanol extract of AP had significant antioxidant effect on five edible oils. However, the study was carried out at a single dose and lacked concentration dependence [122]. As a common TCM, AP has high safety and is widely distributed in various provinces of China. If further study is performed on its antioxidant activity, it is considered possible to make it a natural antioxidant.

### 7.6. Antitumor Effect

Zou et al. studied different concentrations of AP extracts at the cellular level and found that AP inhibited proliferation of human liver cancer cell lines (SMMC-7721) and human umbilical vein endothelial cells (HMVECs) in a dose-dependent manner, with an IC50 value of 1.59 mg/ml. Under the inhibition of 20% of SMMC-7721, the inhibition of HMVECs was more than 50%, showing that there was a significant dose difference between the two cell lines [96]. Lin et al. showed that psoralen, bergapten, xanthotoxin, umbelliferone, osthol, isoimperatorin, and other compounds in AP had antitumor effects, and their antitumor mechanisms may be related to inducing tumor cell apoptosis, inhibiting tumor cell DNA replication, inhibiting tumor cell multidrug resistance, and inhibiting tumor cell metastasis [4,123]. However, this was only the study on the antitumor mechanism of monomer compounds, not AP. And, few studies had conducted comparative studies of the antitumor effects of the crude AP extract and specific compounds in AP. In the future, scholars should also screen out the compounds whose antitumor effects of AP play a dominant role and strengthen their studies on cell and animal levels.

### 7.7. Others

The study by Dan *et al.* showed that chloroform, petroleum ether, and ethyl acetate extracts of AP had significant antigastric ulcer effects [98]. Psoralen-derived compounds, such as bergapten, xanthotoxin, and other compounds, could cause solar dermatitis in humans. Although some experiments had shown that AP has antigastric ulcer and sensitization effects, its mechanism had not been elucidated.

## 8. Toxicity

*Bielu* records “AP as sweet, lukewarm, and nontoxic.” The 2015 edition of the *Chinese Pharmacopoeia* stipulates that the safe dose of AP is 3–10 g per day for adults. Studies have shown that AP contains xanthotoxin, bergapten, psoralen, and osthol, all of which have a certain degree of photosensitivity. Taking too much AP may cause a range of toxic effects. It was recorded in *the Chinese Materia Medica* (中华本草) that the LD50 of intramuscular injection of xanthotoxin in rats was 160 mg/kg and the LD50 of bergapten was 945 mg/kg. 400 mg/kg xanthotoxin could cause death and adrenal hemorrhage in guinea pigs. 200–300 mg/kg xanthotoxin could cause liver turbidity, fatty change, acute hemorrhagic necrosis, severe renal congestion, and hematuria. Continuous administration of 1–2 mg/kg xanthotoxin for 5 months could lead to liver necrosis. The LD50 of osthol intraperitoneally injected in mice was 16 mg/kg [25].

Another report was on two cases of AP poisoning: (1) a male, 9 years old, accidentally ate AP about 100 g, and 1 h after eating, he began to vomit, was irritable and incoherent, had whole bodies convulsions, went into coma, and finally died; (2) a male, 7 years old, accidentally ate AP about 50 g, and 1 h after eating, he began to vomit and had restlessness. Physical examination revealed the following: body temperature was 36.2 °C, pulse was 82 times/min, blood pressure was 100/80, dilated double pupils, slow response to light, arrhythmia, peripheral blood leukocytes were 20400, neutrophils were 83%, and lymphocytes were 17% [124].

Kunming mice were used for the acute toxicity test, and the results showed that 10 minutes after oral administration of large dose (15.80 g/kg), the mice developed symptoms of poisoning such as tail tip cyanosis, restlessness, and accelerated breathing, and some of the mice died due to respiratory failure, LD50 was 7.35 ± 0.62 g/kg. Long-term toxicity tests were conducted on Wistar rats and hybrid dogs. The results showed that 10 min after oral administration of large dose (825 mg/kg), the rats developed symptoms of poisoning such as tail tip cyanosis, restlessness, and rapid breathing. However, the poisoning symptoms disappeared 30 days after oral administration. The growth rate of the high-dose group was slower than that of the control group (*P* < 0.01). Pathological examination revealed gastric flatulence and mucosal edema in some rats. Under a light microscope, hyaline bodies were observed in a few rats and dogs (138 mg/kg) in the high-dose group [125].

At present, there are few studies on the toxicity of AP, and future scholars should focus on the toxicity of AP. In order to ensure the safety and efficacy of AP, toxicity evaluation can provide guidance for clinical drug efficacy and patient safety [126].

## 9. Conclusion and Prospect

AP, as a famous TCM, with complex chemical components and extensive pharmacological effects, has been widely used in China for more than 2000 years. Scholars have done a lot of research on AP. So far, more than 120 coumarin compounds and 220 volatile oils compounds had been isolated from AP. It has also developed from the initial treatment of rheumatism alone to the modern prevention and treatment of tumor, cardiovascular and cerebrovascular diseases, and various arthritis diseases, which provides great help for its development and clinical application. However, there is still a lot of work to be done on the development and utilization of AP.

First, for the quality control of AP, the species of AP are complex, and the species of AP collected in different regions are not the same, and the identification of appearance and character alone is not perfect. Therefore, further research and demonstration are needed to identify authentic AP products, substitutes, and differences in their efficacy. The *Chinese Pharmacopoeia* takes the dry root of *Angelica pubescens* Maxim, f. *biserrata* Shan et Yuan as authentic AP product, which only takes the content of osthol and columbianadin as the quality control standard of AP, which lacks reliability because there is no research to prove that these two compounds are bioactive substances of AP. And, we should analyze the whole characteristics of AP to find out the most effective active ingredients and establish the quality control standard of AP on this basis.

Second, the processing of TCM has the effect of enhancing effect and reducing poison, which is widely used in the processing of TCM. However, there are no reports on processing AP in modern studies including phytochemistry, pharmacology, pharmacodynamics, and pharmaceutical analysis. Studies have shown that APR enters the kidney meridian and bladder meridian. It has a better pharmacological effect among TCMs which enters the kidney meridian after being baked with salt or wine. We speculate that AP can induce the decline of the body after salt roasting, which can better treat knee osteoarthritis and increase the analgesic function of the drug. And, AP after wine roasting can induce medicine to go to the top of the body, relieve headache better, and enhance the effect of blood circulation and activating collaterals. Therefore, it is necessary to study the processing products of AP, which can also fill the gap of AP processing.

Third, the research on the pharmacodynamic substances of AP is not thorough, and the mechanism, target, and pathway of the action of the active ingredients on the disease have not been scientifically clarified. Modern scholars can consider using network pharmacology, data mining, and virtual screening techniques to predict the chemical components and pharmacological activity related to the target, receptor, and pathway and verify through pharmacological experiments. In addition, new data mining methods can be proposed to screen herbs with similar effects, so as to reduce the development cost of new drugs by using natural drugs more effectively. And, most of the research studies focus on preliminary experiments in animals, and more *in vitro* studies at the cell level and more comprehensive clinical application are needed to further confirm its pharmacological mechanism. It is believed that with the innovation of the theories of phytochemistry, pharmacology, pharmacodynamics, etc., and the technical means of chromatography-mass spectrometry, metabonomics, etc., a more scientific and reasonable quality control standard will be formulated, and the mechanism, target, and channel of its effective components will also be clarified, which will make up for the shortage of modern application of TCMs.

Fourth, in order to ensure safety and effectiveness, toxicity assessment can provide guidance for clinical efficacy and patient safety. At the same time, toxicity evaluation is also a prerequisite for the development of new drugs. However, there are few reports on the toxicity evaluation and the absorption, distribution, metabolism, and excretion of the main active components of AP *in vivo*. Future scholars should also study the toxicity and pharmacokinetic characteristics of the compounds in AP, so as to provide experimental basis for AP to become a new drug.

AP, as a TCM, plays an important role in maintaining human health. We hope that this paper can provide more understanding of AP and provide guidance for its future research. We also firmly believe that with the continuous deepening of the research on AP, its application can be extended from the clinical medicine field to health products, cosmetics, and other fields, and occupy a place in the world market.

## Figures and Tables

**Table 1:** The prescription composition, dosage form, and efficacy of AP.

Preparation name	Main compositions	AP dosage	Formulation	Traditional and clinical uses	Reference
Duhuo Jisheng Wan Duhuo Jisheng Heji Duhuo Jisheng Tang	Radix Angelicae Pubescentis	54 g	Pill mixture decoction	Nourish blood, relax tendon, dispel wind, eliminate dampness, and nourish liver and kidney. It is used to treat arthralgia and lumbago and knee pain.	*Chinese Pharmacopoeia*
	Taxilli Herba	98 g			
	Rehmanniae Radix	125 g			
	Achyranthis Bidentatae Radix				
	Asari Radix et Rhizoma				
	Gentianae Macrophyllae Radix				
	Poria				
	Cinnamomi Cortex				
	Saposhnikoviae Radix				
	Chuanxiong Rhizoma				
	Codonopsis Radix				
	Glycyrrhizae Radix et Rhizoma				
	Angelicae Sinensis Radix				
	Eucommiae Cortex				
	Paeoniae Radix Alba				
Duhuo Jiu	Radix Angelicae Pubescentis	200 g	Wine	Treatment of rheumatic joint pain.	*Qianjinfang*
	Saposhnikoviae Radix				
	Typhonii Rhizoma				
Duhuo Cangzhu Tang	Radix Angelicae Pubescentis	Unknown	Decoction	Dissipate cold, relieve pain.	*Zhengyinmaizhi*
	Atractylodis Rhizoma				
	Saposhnikoviae Radix				
	Asari Radix et Rhizoma				
	Chuanxiong Rhizoma				
Duhuo Tang	Radix Angelicae Pubescentis	25 g	Decoction	Treatment of chronic limb pain.	*Huoyouxinshu*
	Angelicae Sinensis Radix				
	Atractylodis Macrocephalae Rhizoma				
	Astragali Radix				
	Achyranthis Bidentatae Radix				
Duhuozi Tang Yiwu Duhuo Tang Duhuo Xixin Tang	Radix Angelicae Pubescentis	500 g	Decoction	Tonify and replenish blood and kidney.	*Qianjinfang*
	Radix Angelicae Pubescentis	150 g	Decoction	Treatment of postpartum stroke.	*Xiaopinfang*
	Radix Angelicae Pubescentis		Decoction	Treat headaches caused by coldness.	*Zhengyinmaizhi*
	Asari Radix et Rhizoma				
	Chuanxiong Rhizoma				
	Gentianae Macrophyllae Radix				
	Rehmanniae Radix				
	Saposhnikoviae Radix				
Duhuo San	Radix Angelicae Pubescentis	50 g	Pulvis	Treatment of all types of tumors.	*Pujifang*
	Scutellariae Radix				
	Angelicae Sinensis Radix				
	Chuanxiong Rhizoma				
	Rhei Radix et Rhizoma				
	Paeoniae Radix Rubra				
Tianhe Zhuifeng Gao	Asari Radix et Rhizoma	Unknown	Ointment	Warm channel and expelling cold, disperse wind and eliminate dampness, and activate blood and relieve pain. Used for joint pain and limb numbness caused by wind, cold, dampness, and blood stasis.	*Chinese Pharmacopoeia*
	Paeoniae Radix Alba				
	Radix Angelicae Pubescentis				
	Paeoniae Radix Rubra				
	Angelicae Sinensis Radix				
	Achyranthis Bidentatae Radix				
Tianma Wan	Gastrodiae Rhizoma	50 g	Pill	Expel wind and eliminate dampness, dredge collateral and relieve pain, tonify and replenish liver and kidney. Used for rheumatic stasis resulting in numbness of limbs and pain in waist and leg.	*Chinese Pharmacopoeia*
	Radix Angelicae Pubescentis				
	Achyranthis Bidentatae Radix				
	Rehmanniae Radix				
	Eucommiae Cortex				
	Angelicae Sinensis Radix				
Tianma Qufeng Bupian	Rehmanniae Radix	50 g	Tablet	Warm kidney nourishing the liver, dispel wind pain. Used for dizziness, tinnitus, joint pain, pain in waist and knee, and chills caused by liver and kidney loss, rheumatism and collateral penetration.	*Chinese Pharmacopoeia*
	Angelicae Sinensis Radix				
	Radix Angelicae Pubescentis				
	Eucommiae Cortex				
	Achyranthis Bidentatae Radix				
	Poria				
Zhonghua Dieda Wan	Radix Angelicae Pubescentis	76.8 g	Pill	Detumescence and analgesia, relax tendons and activate collaterals, hemostatic myosheng, invigorate blood, and remove blood stasis. Used for bruising bones, old and new bruising, trauma bleeding, rheumatic bruising.	*Chinese Pharmacopoeia*
	Cyperi Rhizoma				
	Cinnamomi Ramulus				
	Achyranthis Bidentatae Radix				
	Eucommiae Cortex				
	Atractylodis Rhizoma				
Zhengtian Wan Zhengtian Jiaonang	Angelicae Sinensis Radix	102 g	Pill, capsule	Expel wind and activate blood, nourish blood and tonic liver, dredge collateral and relieve pain. It is used for migraines, tension headache, nerve headache, cervical spondylosis type headache, and premenstrual headache caused by external wind evil, blood stasis, blood loss of nutrition, hyperactivity of liver yang.	*Chinese Pharmacopoeia*
	Paeoniae Radix Alba				
	Saposhnikoviae Radix				
	Asari Radix et Rhizoma				
	Radix Angelicae Pubescentis				
	Ephedrae Herba				
Zhuanggu Guanjie Wan	Cibotii Rhizoma	Unknown	Pill	Tonify the liver and kidney, nourish blood and promote blood circulation, relax tendons and collaterals, regulate qi and relieve pain. Used for the treatment of arthritis, lumbar muscle strain, joint swelling, pain, numbness, limited movement.	*Chinese Pharmacopoeia*
	Radix Angelicae Pubescentis				
	Dipsaci Radix				
	Taxilli Herba				
	Rehmanniae Radix				
	Psoraleae Frsuctus				
Wangbi Pian Wangbi Keli	Rehmanniae Radix	Unknown	Tablet	Tonify and replenish liver and kidney, strengthen tendons and bones. Disperse wind and dehumidification, dredge collateral and relax tendon. Used to treat rheumatoid arthritis.	*Chinese Pharmacopoeia*
	Dipsaci Radix				
	Radix Angelicae Pubescentis				
	Saposhnikoviae Radix				
	Rehmanniae Radix				
	Clematidis Radix et Rhizoma				
Guogong Jiu	Angelicae Sinensis Radix	Unknown	Wine	Disperse wind and dehumidification, relax sinew and dredge collateral. Used for the treatment of joint pain, adverse flexion and extension, hand and foot numbness, pain in waist and leg. It is also used for hemiplegia and askew caused by imbalance between channels and collaterals.	*Chinese Pharmacopoeia*
	Achyranthis Bidentatae Radix				
	Radix Angelicae Pubescentis				
	Paeoniae Radix Alba				
	Psoraleae Fructus				
	Saposhnikoviae Radix				
Baidu San	Codonopsis Radix	100 g	Pulvis	Relieving exterior syndrome by diaphoresis, disperse wind and dehumidification. Used to treat fever, headache, sore limbs, stuffy nose, cough and phlegm.	*Chinese Pharmacopoeia*
	Aurantii Fructus				
	Chuanxiong Rhizoma				
	Radix Angelicae Pubescentis				
	Peucedani Radix				
	Bupleuri Radix				
Goupi Gao	Saposhnikoviae Radix	20 g	Ointment	Disperse wind and dissipate cold, activate blood and relieve pain. Used for the treatment of limb numbness, lumbago and leg pain, or bruise injury, local swelling and pain.	*Chinese Pharmacopoeia*
	Ephedrae Herba				
	Angelicae Sinensis Radix				
	Dipsaci Radix				
	Paeoniae Radix Alba				
	Radix Angelicae Pubescentis				
Fufang Xiatianwu Pian	AGKISTRODON	Unknown	Tablet	Disperse wind and dehumidification, relax the tendons and collaterals, blood circulation and pain. It is used for the treatment of joint swelling pain, limb numbness, adverse flexion and rheumatoid arthritis, sciatica caused by obstruction of meridians and collaterals and rheumatic stasis block, sequelae of cerebral thrombosis and poliomyelitis.	*Chinese Pharmacopoeia*
	Radix Angelicae Pubescentis				
	Clematidis Radix et Rhizoma				
	Salviae Miltiorrhizae Radix et Rhizoma				
	Achyranthis Bidentatae Radix				
Huoxue Zhitong Gao	Paeoniae Radix Alba	5.4 g	Ointment	Activate blood and relieve the pain. Used for the treatment of muscle and bone pain, muscle paralysis, resolve phlegm, joint pain.	*Chinese Pharmacopoeia*
	Citri Reticulatae Pericarpium				
	Angelicae Sinensis Radix				
	Asari Radix et Rhizoma				
	Schizonepetae Herba				
	Radix Angelicae Pubescentis				
Qufeng Zhitong Wan Qufeng Zhitong Pian Qufeng Zhitong Jiaonang	Dipsaci Radix	83 g	Pill, tablet, capsule	Disperse wind and dehumidification, tonify and replenish liver and kidney, strengthening tendons and bones used for the treatment of joint swelling, lumbago and knee pain, limb numbness.	*Chinese Pharmacopoeia*
	Clematidis Radix et Rhizoma				
	Radix Angelicae Pubescentis				
Tongbi Jiaonang	Astragali Radix	4.42 g	Capsule	Disperse wind and dehumidification, activate blood and dredge collateral, dissipate cold and relieve pain, nourish and activate blood. Used for the treatment of cold joint pain, flexion and extension, rheumatoid arthritis.	*Chinese Pharmacopoeia*
	Angelicae Sinensis Radix				
	Radix Angelicae Pubescentis				
	Saposhnikoviae Radix				
	Typhonii Rhizoma				
	Corydalis Rhizoma				
Jisheng Zhuifeng Jiu	Radix Angelicae Pubescentis	Unknown	Wine	Tonify and replenish liver and kidney, disperse wind and dehumidification, relieve pain. For the treatment of liver and kidney two deficient, wind cold dampness bi, waist and knee cold pain, flexion and extension, rheumatoid arthritis, lumbar muscle strain, injury.	*Chinese Pharmacopoeia*
	Achyranthis Bidentatae Radix				
	Saposhnikoviae Radix				
	Asari Radix et Rhizoma				
	Angelicae Sinensis Radix				
	Poria				
Shujin Wan	Strychni Semen	6 g	Pill	Disperse wind and dehumidification, relax tendon and activate blood. For the treatment of wind, cold and dampness, numbness of limbs, muscle and bone pain, walking difficult.	*Chinese Pharmacopoeia*
	Ephedrae Herba				
	Radix Angelicae Pubescentis				
	Saposhnikoviae Radix				
	Eucommiae Cortex				
	Dipsaci Radix				
Shujin Huoluo Jiu	Taxilli Herba	30 g	Wine	Disperse wind and dehumidification, activate blood and dredge collateral, tonify yin and promote fluid production. Used for adverse flexion and extension, limb numbness, joint pain caused by yin deficiency, rheumatism blocking collateral, blood stasis.	*Chinese Pharmacopoeia*
	Achyranthis Bidentatae Radix				
	Angelicae Sinensis Radix				
	Chuanxiong Rhizoma				
	Radix Angelicae Pubescentis				
	Saposhnikoviae Radix				
Shufeng Dingtong Wan	Strychni Semen	30 g	Pill	Disperse wind and dissipate cold, activate blood and relieve pain. Used for joint pain, cold pain, stabbing pain, waist and leg pain and limb numbness caused by wind, cold and wet closure, and blood stasis. Local swelling caused by a bruise.	*Chinese Pharmacopoeia*
	Achyranthis Bidentatae Radix				
	Saposhnikoviae Radix				
	Radix Angelicae Pubescentis				
	Ephedrae Herba				
	Eucommiae cortex				
Yaobitong Jiaonang	Notoginseng Radix et Rhizoma	Unknown	Capsule	Activate blood and resolve stasis, dispel wind and eliminate dampness, move qi and stop pain. Used for lumbago caused by blood stasis and qi stagnation.	*Chinese Pharmacopoeia*
	Chuanxiong Rhizoma				
	Corydalis Rhizoma				
	Paeoniae Radix Alba				
	Achyranthis Bidentatae Radix				
	Radix Angelicae Pubescentis				
Shexiang Qutong Qiwuji Shexiang Qutong Chaji	Moschus	1 g	Aerosol liniment	Activate blood and resolve stasis, relax sinew and activate collateral, eliminate swelling and relieve pain. Used for all kinds of fall injury, blood stasis swelling pain, rheumatism stasis, joint pain.	*Chinese Pharmacopoeia*
	Carthami Flos				
	Radix Angelicae Pubescentis				
	Rehmanniae Radix				
	Notoginseng Radix et Rhizoma				

**Table 2:** The coumarin compounds of AP.

No.	Compounds	Reference
1	Acaculetindimethylether	[43]
2	Angelicae lactone aldehyde-6-formyl-7-methoxycoumarin	[43]
3	Allimperatorin	[43]
4	Angelicin	[43]
5	Angelicone	[43]
6	Angelidiol	[43]
7	Angelin	[43]
8	Angelitriol	[43]
9	Angelmarin	[43]
10	Angelol A	[43]
11	Angelol B	[43]
12	Angelol C	[43]
13	Angelol D	[43]
14	Angelol E	[43]
15	Angelol F	[43]
16	Angelol G	[43]
17	Angelol H	[43]
18	Angelol I	[43]
19	Angelol J	[43]
20	Angelol K	[43]
21	Angelol L	[43]
22	Angenomalin	[44]
23	Angepubebisin	[43]
24	Anhydrobyakangelin	[43]
25	Anpubesol	[43]
26	Apaensin	[43]
27	Apiosylckimmin	[43]
28	Apterin	[43]
29	Bergapten	[43]
30	Bergaptol	[43]
31	Bisabolangelone	[45]
32	Byakangelicin	[43]
33	Byakangelicol	[43]
34	Cnidilin	[43]
35	Columbianetin	[43]
36	Columbianetin	[43]
37	Columbianetin acetate	[43]
38	Columbianetin-*β*-D-glucopyranoside	[43]
39	Columbianetin propionate	[45]
40	Columbianadin	[43]
41	Coumurrayin	[43]
42	Dehydra-angelol A	[39]
43	Dehydra-angelol B	[39]
44	Dehydra-angelol C	[39]
45	2-Deoxymeranzin hydrate	[44]
46	Dihydrocolumbianadin	[45]
47	Ferulin	[41]
48	8-(3-Hydroxylsoval-croyl-5,7-dimethoxycoumarin)	[43]
49	5-(2-Hydroxy-3-methoxy-3-methylbutoxy-psoralen)	[43]
50	Imperatorin	[43]
51	Isoangenomalin	[45]
52	Isoanglol	[43]
53	Isobergapten	[43]
54	Isoimperatorin	[43]
55	Isooxypencedanine	[43]
56	Isopimpinellin	[43]
57	Isopsoralen	[43]
58	Marmesinin	[43]
59	Meranzin hydrate	[43]
60	7-Methoxy-8-sene-cloylcoumarin	[43]
61	Neobyakangelicol	[43]
62	Nodakenetin	[45]
63	Nodakenin	[43]
64	Osthenol	[43]
65	Osthol	[43]
66	Oxypeucedanin	[43]
67	Oxypeucedania hydrate	[43]
68	Pabulenol	[43]
69	Peucedanol	[43]
70	Psoralen	[43]
71	Scopoletin	[43]
72	sec-O-Acetyl-byakangelicin	[43]
73	sec-O-*β*-D-Glucopy-ranosyl-(R)-byakangelicin	[41]
74	tert-O-*β*-D-Glucopy-ranosyl-(R)-byakangelicin	[41]
75	Ulopterol	[43]
76	Umbelliferone	[43]
77	Umbelliprenin	[43]
78	Vaginidiol	[43]
79	Xanthotoxin	[43]

**Table 3:** The volatile oils compounds of AP.

No.	Compounds	Reference
1	Acorenone	[61]
2	Aristolene	[49]
3	Benzene-1-methyl-2-(1-methylethyl)	[49]
4	Benzene-2-methoxy-4-methyl-1-(1-methylethyl)	[49]
5	Benzene-1,2,4-trimethoxy-5-(1-proprnyl))	[49]
6	*α*-Bergamotene	[43]
7	Bicyclo-[3, 10]-hexan-3-ol	[49]
8	Bicycol [7, 2, 0]undec-4-ene, 4,11,11-trimethyl-8-methylene	[43]
9	*α*-Bisabolene	[43]
10	*β*-Bisabolene	[43]
11	Camphene	[44]
12	6-Camphenone	[49]
13	3-Carene	[48]
14	Cedrol	[49]
15	*α*-Copaene	[43]
16	p-Cresol	[43]
17	Cyclohexene	[49]
18	*α*-Cyclohexyl decane	[43]
19	1,3-Cyclohexadiene-5-(1,5-dimethyl-4-hexenyl-2-methyl	[43]
20	(+)-Cycloisosativene	[49]
21	2-Cyclopropen-l-one,2,3-diphenyl	[43]
22	Decylacetate	[44]
23	[s-(R, s)]-3-(1,5 Dimethyl-4-hexene)-6-methylene-cyclohexene	[44]
24	3,6-Dimethyloctane	[49]
25	2,2-Dimethyl-8-oxo-3,4-dihydro-2H,8H-pyrano[3,2-g] chromen-3-yl ester	[50]
26	8,8-Dimethyl-2-oxo-2,8,9,10-tetrahydropyrano(2,3-f)chromene-9,10-diylbis	[50]
27	5,6-Dimethyl-3a,4,7,7a-H	[43]
28	*α*-Elemene	[43]
29	*β*-Elemene	[43]
30	Elemol	[50]
31	Elixene	[43]
32	Eremophilene	[43]
33	[1S-(1*α*, 2*β*, 4*β*)]-1-Ethenyl-1-methyl-2,4-bis-(1-methyethenyl)-cyclohexane	[44]
34	*α*-Funebrene	[43]
35	Globulol	[49]
36	Guaiol	[43]
37	*β*-Gurjunene	[50]
38	(Z)-7-Hexadecenal	[49]
39	1-Hexadecanol	[50]
40	3-Hexanol	[44]
41	Humulene	[43]
42	4-Hydroxy-3-methylacetophenone	[47]
43	1,3-Isobenzafurandione,3a,4,7,7a-tetrahydro-5,6-dimethyl	[51]
44	Isopropy-4-methylene-7-methyl-1, 2, 3,4, 4A, 5. 6, 8A-octahydronaphthalene	[43]
45	Isosericenine	[43]
46	Ledol	[49]
47	Leecanoic acid,2,4,6-trirnethyl-methyl ester	[43]
48	Lignoceric acid	[51]
49	*d*-Limonene	[48]
50	4-Longifolene	[49]
51	*α*-Longipinene	[43]
52	4-Methoxy-6-(2-propenyl)-1,3-benzodioxole	[49]
53	4-Methoxyacetophenone	[43]
54	8-Methyl-1-decene	[44]
55	3-Methyl-but-2-enoic acid	[50]
56	4-Methylclohexanone	[43]
57	1-Methyl-1,4-cyclohexadiene	[44]
58	4-Methylene-1-(1-methylethyl)-bicyclo [3, 1, 0] hexane	[44]
59	4-Methylene-1-(1-methylethyl)-bicyclo [3, 1, 0] hex-2-ene	[44]
60	4-Methylene-1,5,5-trimethylcyclohecene	[44]
61	4,4′-Methylenebis[2,3,5,6-tetremethyl] phenol	[43]
62	2-[1-Methylethyl-methylcarbam ate] phenol	[49]
63	2-Methyl-1-methylene-3-(1-methylethenyl)-cyclopentane	[44]
64	2-Methyl-5-(1-methylethyl)-phenol	[49]
65	3-Methylnolane	[43]
66	2-Methyloctane	[43]
67	o-Methylphenol	[49]
68	9-Methyl-1-undecene	[49]
69	10-Methyl-1-undecene	[49]
70	Myrcene	[43]
71	Myrtenal	[43]
72	Naphthalene-1,2,3,4,4*α*,5,6,8*α*-octahydro-7-methyl-4-methylene-1-(1-methylethyl)-(1,4a,8a)	[43]
73	Naphthalenone	[49]
74	Nerolidol	[43]
75	n-Nonane	[43]
76	n-Octane	[43]
77	n-Undecane	[43]
78	9,12-Octadecadienoic acid methyl ester	[49]
79	o-Cymene	[47]
80	Osthol	[43]
81	Oxacyclohexadecan-*α*-one	[43]
82	*γ*-Patchoulene	[43]
83	p-Cresol	[51]
84	p-Cymene	[9]
85	7, 10-Pentadecadiynoic acid	[43]
86	1-Penten-3-one thyl vinyl ketone	[43]
87	Pentylbenzene	[49]
88	Peucedanol	[43]
89	Phenol,4-(1,1-dimethylethyl)-2-methyl	[43]
90	*α*-Phellandrene	[43]
91	*α*-Pinene	[43]
92	*β*-Pinene	[43]
93	*α*-Selinene	[47]
94	*β*-Sesquiphellandrene	[47]
95	Sylvestrene	[43]
96	*α*-Terpinene	[43]
97	1-Tetradecene	[43]
98	1,5,5,8-Tetramethyl-12-oxabicyclo [9, 1, 0] dodeca-3,7-diene	[44]
99	Thalic acid diisobuthyl ester	[49]
100	Thymol	[43]
101	Toluene	[49]
102	trans-p-Menth-2-en-7-ol	[51]
103	3, 7, 11-Tridecatrienoic acid,4,8,12-trimethl-ester[Z,E]	[43]
104	1,7,7-Trimethylbicyclo-[2.2.1]-hept-2-yl-acetate	[50]
105	2,4,6-Trimethylmethyl ester-decanoic acid	[43]
106	1,3,3-Trimethyl-tricyclo[2.2.1.02,6] heptane	[44]
107	7*α*-Twtrahydro-5,6-dimethyl	[43]
108	Ulopterol	[43]
109	Zingiberene	[49]

**Table 4:** The organic acid compounds of AP.

No.	Compounds	Reference
1	Caffeic acid	[43]
2	3-O-Caffeoylquinic acid	[71]
3	4-O-Caffeoylquinic acid	[71]
4	5-O-Caffeoylquinic acid	[71]
5	Ferulic acid	[71]
6	1,5-Dicaffeoylquinic acid	[71]
7	3,4-Dicaffeoylquinic acid	[71]
8	3,5-Dicaffeoylquinic acid	[71]
9	4,5-Dicaffeoylquinic acid	[71]
10	Isoferulic acid	[44]
11	[2,3,4,9-Tetrahydro-1H-pyrido-(3,4-b)]-indole-3-(ar-boxy-lic acid)	[71]
12	3-O-trans-Coumaroylquinic acid	[44]
13	3-O-trans-Feruloylquinic acid	[44]

**Table 5:** The pharmacology of AP.

Effects	Extract/compound dose	Animal/cell line	Study design	Control	Mechanism/results	Ref
Anti-inflammatory effect	1.5 g/kg (40%, 60%, 80%) ethanol extract solution	Xylene-induced mice ears edema	*In vivo*	Aspirin	By inhibiting the activity of NAAA, intracellular biological activity was increased and the level of proinflammatory factors was decreased.	[93]
		Egg white-induced mice pettitoes swelling				
		Tampon granulation-induced mice swelling				
	0.4 mL 50% ethanol extract solution	Xylene-induced mice ears edema	*In vivo*	Aspirin	It plays an anti-inflammatory role by reducing the levels of inflammatory factors such as MDA and TNF-*α*.	[92]
		Carrageenan-induced mice swelling				
	0.15 and 0.29 g/kg volatile oil of AP	Egg white-induced rat pettitoes swelling	*In vivo*	Hexadecadrol	It plays an anti-inflammatory role by inhibiting the release of inflammatory factors such as 5-HT.	[94]
	AP 95% ethanol extract solution (0.1 g/L, 1 g/L, 10 g/L)	LPS-induced rat peritoneal macrophages	*In vitro*	Celecoxib	AP inhibited COX-1 and COX-2 in varying degrees, and there was a dose-response relationship.	[99]
				Indomethacin		
				DMSO		
	Volatile oil of AP (10 mg/L, 50 mg/L, 250 mg/L)	LPS-induced RAW 264.7 cell	*In vitro*	None	By inhibiting the hydrolysis activity of NAAA and increase the level of N-PEA, downregulating the expression of TNF-*α*, iNOS, IL-6 mRNA, and inhibiting the release of TNF-*α* and NO.	[100]
	Angesesquid A and angesesquid B	Primary chondrocytes of rat intervertebral disc	*In vitro*	None	Inflammation is inhibited by inhibiting the release of NO.	[101]
Analgesic	1.5 g/kg (40%, 60%, 80%) ethanol extract solution	Acetic acid-induced and tail-immersion-induced writhing	*In vivo*	Aspirin	By inhibiting the activity of NAAA, the intracellular biological activity was increased and the level of inflammatory factors was reduced to play an analgesic role.	[93]
	0.4 mL 50% ethanol extract solution	Hot plate, acetic acid, and formalin-induced pain	*In vivo*	Aspirin	By inhibiting the level of MDA and inflammatory factors was reduced to play an analgesic role.	[92]
	0.15 and 0.29 g/kg volatile oil of AP	Hot plate, acetic acid -induced mice pain	*In vivo*	Aspirin	AP has an analgesic effect similar to that of nSAID rather than narcotic analgesics.	[94]
				Morphine hydrochloride		
	Coumarins in AP	Spared nerve injury model rat	*In vivo*	Morphine	AP has the analgesic effect mainly related to reducing the concentration of proinflammatory cytokines of TNF-*α*, IL-1 *β*, and IL-6 and reducing the expression of TRPV1 and perk in the damaged neurons.	[94]
Cardiovascular effect	0.4 g/kg, 1.0 g/kg AP ethanol extract	ADP-induced mice platelet aggregation	*In vivo*	Black	AP alcohol extract can shorten the thrombus length and prolong the tail hemorrhage time of mice to inhibit ADP-induced platelet aggregation in mice.	[103]
	10 mg/kg GABA	Multiple arrhythmia models	*In vivo*	Black	GABA could prolong the start time, reduce the incidence of ventricular tachycardia, shorten the duration of ventricular tachycardia, reduce the mortality of ventricular fibrillation, reduce APA and APO50 and APO90.	[104]
	AP ethanol extract	Patients with vertigo	*In vivo*	Compound Danshen	By reducing the whole blood viscosity, plasma viscosity and red blood cell aggregation index of vertigo patients, increasing the cerebral blood flow speed to play the role of promoting blood circulation and removing stasis.	[105]
	1.28 g/ml AP 95% ethanol extract	Thoracic aortic rings in rats	*In vitro*	CaCl2	AP had a good diastolic effect on vasoconstriction caused by PE and KCl, and its mechanism was related to the influx of CaCl_2_.	[106]
	3.75–30 *μ*g/ml AP 95% ethanol extract and osthol	Human umbilical vein endothelial cells	*In vitro*	None	Inhibition of angiogenesis by stagnation of endothelial cycle mainly in G0-G1.	[11]
		Human colon cancer cell				
Neuroprotective effect	AP 90% ethanol extract (2.7 g/kg/d, 8.1 g/kg/d, 24.3 g/kg/d)	D-Galactose-induced mice aging model	*In vivo*	Model	AP's delay of brain aging is associated with anti-free radical peroxide damage, reduction of immunosuppression of arachidonic acid metabolites, and antagonism of brain inflammation.	[95]
	12 ml/kg/d AP alcohol extract or AP water extract	Aged mice	*In vivo*	Black	AP could reduce the content of MDA content and the deletion of DNA fragment in natural aging mice.	[96]
	Coumarin in AP with 22.05 mg/kg/d, 66.15 mg/kg/d, 198.45 mg/kg/d	Lactacystin-induced PD rat model	*In vivo*	Madopar, ibuprofen	Inhibition of lipid peroxidation in serum and brain tissue, increase of antioxidant enzyme activity, and decrease of excitatory amino acid Glu content in serum and brain tissue.	[107]
	Coumarin in AP with 3 g/kg/d	Lactacystin-induced PD rat model	*In vivo*	Madopar, celecoxib	Coumarin in AP and AP may protect PD by inhibiting endoplasmic reticulum stress.	[108]
	AP granules 3 g/kg/d					
	Compound AP granules 6.3 g/kg/d					
	AP 90% ethanol extract or water extract (18 g/kg/d)	Mice aged 16 months	*In vivo*	Water	It can improve the activity of mitochondrial respiratory chain enzyme complex I and IV in the brain of aged mice and protect the oxidative damage of mitochondrial DNA in mice.	[109]
	2 ml/mouse AP extract	A*β*-induced rat dementia model	*In vivo*	Indometacin	AP can inhibit the expression of P38 MAPK in rat brain and improve the learning and memory ability of dementia model rats.	[110]
	1 g/kg or 4 g/kg AP ethanol extract	D-Galactose or sodium nitrite-induced Alzheimer's disease mice	*In vivo*	Water	AP can delay the occurrence of Alzheimer's disease by increasing SOD and reducing AChE in brain tissue.	[111]
	20 mg/ml osthol	APP/PS1 double transgenic mice	*In vivo*	None	Osthol can promote the survival of NT-3-BM-NSCs and enhance the cholinergic nerve function in the brain.	[112]
	10–250 mg/ml coumarin in AP	A*β*-induced mice nerve injury model	*In vivo*	None	By promoting the expression of CREB and BDNF, the expression of P-CREB and BDNF is increased to play a neuroprotective role.	[113]
	9, 18, 36 ng/kg AP extract	MOG35-55 peptide fragment-induced mice EVE model	*In vivo*	Prednisone acetate	It can alleviate demyelination injury, inhibit the secretion of proinflammatory cytokines by spleen lymphocytes, and has neuroprotective effect.	[114]
Antibacterial effect	AP petroleum ether extract	Fungus	*In vitro*	None	AP essential oil has different degree of bacteriostatic effect on different fungi.	[115]
	1 g/L AP ethanol extract	*Penicillium italicum*	*In vitro*	None	Isobergamolactone, sphondin, pimpinellin, and isopimpinellin in AP showed antibacterial activity.	[116]
		*Penicillium digitatum*				
		*Colletotrichum gloeosporioides*				
Antioxidation effects	12 ml/kg/d AP alcohol extract or AP water extract	Aged mice	*In vivo*	Black	AP could reduce the level of MDA in the brain tissues of mice.	[96]
	1 g/kg or 4 g/kg AP ethanol extract	D-Galactose or sodium nitrite-induced Alzheimer's disease mice	*In vivo*	Water	AP could delay the occurrence of Alzheimer's disease by increasing the level of SOD.	[111]
	0.4 ml AP 50% ethanol extract	Carrageenan-induced mice swelling	*In vivo*	Aspirin	AP alcohol extract could inhibit the concentration of MDA with the inhibitory rate of 23.49%.	[92]
Antitumor effect	0.3125–10 mg/ml AP aqueous solution	HMVECs	*In vitro*	Cyclophosphamide	AP can inhibit the proliferation of human microvascular endothelial cells. Inhibition of angiogenesis.	[97]
		SMMC-7721				
Antigastric ulcer	Chloroform, petroleum ether, and ethyl acetate extracts of AP	Aspirin-ethanol solution-induced mice gastric ulcer model	*In vivo*	Cimetidine	The fat-soluble part of AP is the effective part against gastric ulcer.	[98]

## Data Availability

All reported or analyzed data in this review are extracted from published articles.
